# Ellagitannin-rich cloudberry inhibits hepatocyte growth factor induced cell migration and phosphatidylinositol 3-kinase/AKT activation in colon carcinoma cells and tumors in Min mice

**DOI:** 10.18632/oncotarget.9724

**Published:** 2016-05-30

**Authors:** Anne-Maria Pajari, Essi Päivärinta, Lassi Paavolainen, Elina Vaara, Tuuli Koivumäki, Ritu Garg, Anu Heiman-Lindh, Marja Mutanen, Varpu Marjomäki, Anne J. Ridley

**Affiliations:** ^1^ Department of Food and Environmental Sciences, Division of Nutrition, University of Helsinki, Helsinki, Finland; ^2^ University College London, Ludwig Institute for Cancer Research, London, UK; ^3^ Department of Biological and Environmental Science / Nanoscience Center, University of Jyväskylä, Jyväskylä, Finland; ^4^ Department of Food and Environmental Sciences, Division of Food Chemistry, University of Helsinki, Helsinki, Finland; ^5^ Randall Division of Cell & Molecular Biophysics, King's College London, New Hunt's House, Guy's Campus, London, UK

**Keywords:** colorectal cancer, cell migration, Met receptor, ellagitannins, Min mouse

## Abstract

Berries have been found to inhibit colon carcinogenesis in animal models, and thus represent a potential source of compounds for prevention and treatment of colorectal cancer. The mechanistic basis for their effects is not well understood. We used human colon carcinoma cells and Min mice to investigate the effects of ellagitannin-rich cloudberry *(Rubus chamaemorus)* extract on cancer cell migration and underlying cell signaling. Intrinsic and hepatocyte growth factor (HGF) -induced cell motility in human HT29 and HCA7 colon carcinoma cells was assessed carrying out cell scattering and scratch wound healing assays using time-lapse microscopy. Activation of Met, AKT, and ERK in cell lines and tumors of cloudberry-fed Min mice were determined using immunoprecipitation, Western blot and immunohistochemical analyses. Cloudberry extract significantly inhibited particularly HGF-induced cancer cell migration in both cell lines. Cloudberry extract inhibited the Met receptor tyrosine phosphorylation by HGF and strongly suppressed HGF-induced AKT and ERK activation in both HT29 and HCA7 cells. Consistently, cloudberry feeding (10% w/w freeze-dried berries in diet for 10 weeks) reduced the level of active AKT and prevented phosphoMet localization at the edges in tumors of Min mice. These results indicate that cloudberry reduces tumor growth and cancer cell motility by inhibiting Met signaling and consequent activation of phosphatidylinositol 3-kinase/AKT *in vitro* and in tumors *in vivo*. As the Met receptor is recognized to be a major target in cancer treatment, our results suggest that dietary phytochemicals may have therapeutic value in reducing cancer progression and metastasis.

## INTRODUCTION

Hepatocyte growth factor (HGF) and its receptor the Met tyrosine kinase induce multiple cellular responses depending on the environmental context, including cell motility, survival, proliferation, morphogenesis and angiogenesis [1, 2]. Met is frequently activated in human cancers and is a target of intensive cancer drug development [3]. Mutations of Met are, however, rare events in cancer and aberrant activation mostly arises from over-expression of the receptor in cancer cells and/or increased secretion of HGF from stromal mesenchymal cells [1, 4]. Binding of HGF to Met leads to dimerization of the receptor and autophosphorylation of several tyrosine residues in the C-terminal tail, including two tyrosine residues (Y1349 and Y1356) that serve as a docking site for adapter signaling molecules such as Grb-2, Gab-1 and Shc [5, 6]. These docking sites activate several key cell signaling pathways including Ras-ERK, phosphatidylinositol 3-kinase (PI3K)/AKT, phospholipase C, Src and STAT3 [4].

In the intestinal tract, HGF is expressed by stromal fibroblasts and fibroblasts within the tumor microenvironment, whereas the Met receptor is expressed by intestinal epithelial cells [7, 8]. Expression of Met is increased at all stages of colon carcinogenesis, including dysplastic ACF, adenomas and invasive carcinomas [8, 9]. Based on the observations that Met expression is localized only to stem and progenitor cells in the intestinal epithelium [10–12], it has been proposed that during tumor formation wild-type Met is over-expressed in cells undergoing aberrant differentiation and retaining stem cell properties, which drives the tumorigenesis and invasive growth [2]. A number of clinical studies have associated Met over-expression with advanced stages of colon cancer and as a predictor of tumor invasion and lymph node metastases [13, 14]. In tumor cell metastasis, the unique ability of activated Met to induce cell motility and migration is considered to play an important role [1].

Colorectal cancer is one of the leading causes of cancer-related deaths in Western societies. So far the most promising chemopreventive agents against colon cancer are pharmaceuticals with undesirable side-effects (*e.g.* COX2 inhibitors) [15]. New strategies to prevent and treat this cancer are therefore required. Berries are a good source of anti-carcinogenic compounds and provide protection against colon tumorigenesis in experimental animal models. For example, freeze-dried black raspberries inhibited intestinal tumorigenesis in *Apc1638^+/−^* and *Muc2^−/−^* mouse models of colorectal cancer [16] and tumor formation in the colon of AOM-treated rats [17]. An anthocyanin mixture from bilberry significantly reduced tumor numbers in the Min mouse [18]. Furthermore, the cancer-preventive effects of berries have recently been tested in humans. Black raspberry powder resulted in regression of rectal polyps when administered to familial adenomatous polyposis (FAP) patients as suppositories [19] and protectively modulated both genetic and epigenetic biomarkers in tissues from sporadic colorectal cancer patients when given orally [20]. In both studies, the treatment period with berries was relatively short and it would be meaningful to study berries as an adjuvant therapy for longer time periods in future.

We studied the effects of bilberry, lingonberry and cloudberry on intestinal tumorigenesis in the Min mouse, an animal model carrying a heterozygous germline mutation in the Apc tumor suppressor gene, similar to human FAP syndrome and the majority of sporadic colorectal cancer cases [21]. Even though the majority of tumors in the Min mouse develop in the distal small intestine and only very few in the colon itself, tumor formation follows the well-established adenoma-carcinoma sequence. We found that all berries resulted in significant reduction in tumor numbers [22]. Cloudberry (*Rubus chamaemorus*), also known as bakeapple or baked-apple berry in Northern America, was by far the most potent chemopreventive berry as it was also able to reduce tumor growth, resulting in over 60% reduction in intestinal tumor burden. Cloudberry occurs naturally throughout the Northern hemisphere, including Scandinavia, Canada and some of the northern states in the US.

In this study, we investigated the anti-carcinogenic activity and underlying molecular mechanisms of cloudberry extract by studying its effects on HGF-induced cell migration using human HT29 and HCA7 colon carcinoma cells in scattering and scratch wound healing assays. We found that cloudberry extract potently inhibited HGF-induced and Met receptor-mediated cancer cell migration and underlying downstream signaling to ERK and AKT *in vitro*. In line with the *in vitro* observations we found that cloudberry reduced AKT activity and localization of phosphorylated Met at the edges in intestinal tumors in Min mice *in vivo*.

## RESULTS AND DISCUSSION

### Cloudberry extract inhibits HGF-induced scattering and scratch wound healing of HT29 and HCA7 colon adenocarcinoma cells

We used scattering and wound healing assays to study the effects of cloudberry extract on cancer cell migration [23, 24]. We chose two genetically well characterized human colon adenocarcinoma cell lines HT29 and HCA7, which represent the two main forms of genetic defects in colorectal cancer etiology, namely chromosomal instability and microsatellite instability. One of the differences between the cell lines is in their status of the tumor suppressor adenomatous polyposis coli (APC) gene: HT29 cells carry two mutated APC alleles whereas HCA7 are wild type for APC [25]. This may be important for migration as APC mutations are associated with defects in cell migration early during experimental colon tumorigenesis [26]. Both cell lines still have an epithelial morphology, similar levels of Met and responsiveness to HGF. The migration assays were carried out by time-lapse microscopy and quantitatively analyzed using an open access, high-throughput image-processing computer platform.

**Figure 1:**
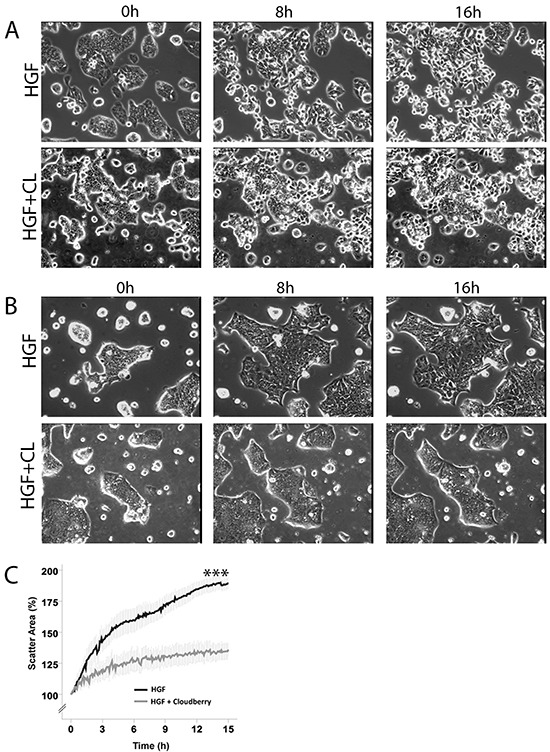
Cloudberry extract inhibits HGF-induced cell scattering in colon adenocarcinoma cells **A.** HT29 and **B.** HCA7 cells were treated either with 40 ng/ml HGF or HGF together with 5 μL/mL cloudberry extract (CL) and followed every 5 min by time-lapse microscopy for 16 hours. Time points 0, 8 h and 16 h are shown in this figure and the movies are available in Supplementary Materials (Movies S1-S4). **C.** Scatter areas were analyzed quantitatively every 5 min by determining the percentage of scatter area as compared to the area at time 0. Results (mean +/− SEM) from 4 independent experiments for HCA7 cells (*P*<0.001 (marked with *** in the figure) HGF vs. HGF+Cloudberry treatment).

**Figure 2:**
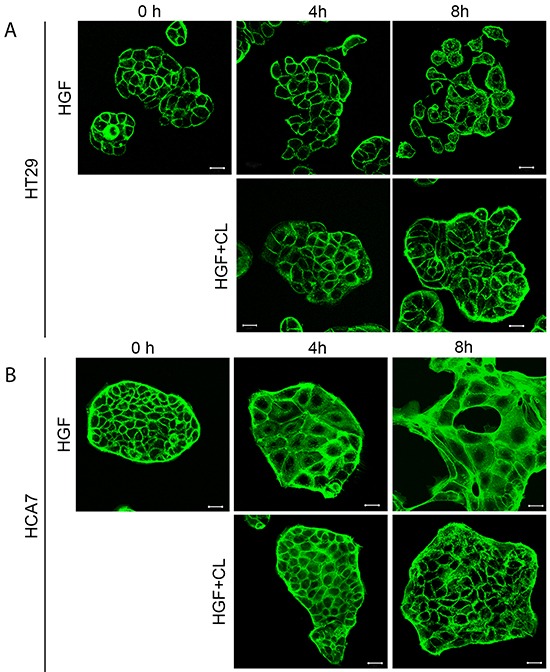
Cloudberry extract prevents HGF-induced morphological changes in colon adenocarcinoma cells **A.** HT29 and **B.** HCA7 cells on glass cover slips were treated with 40 ng/ml HGF or HGF together with 5 μL/mL cloudberry extract (CL) for 0 h, 4 h and 8 h, fixed and stained for actin filaments with FITC-phalloidin and imaged by confocal microscopy. Bars, 25 μm.

Both cell lines showed large morphological changes in response to HGF treatment (40 ng/mL) when followed for 16 hours by time-lapse microscopy (Figure 1, Supplementary Movies S1 and S2). These morphological changes were clearly visible after 4 hours of HGF treatment as demonstrated by confocal images of actin filaments in HT29 and HCA7 cells (Figure 2). HT29 cells scattered in many respects similarly to the well-established scattering of MDCK cells which involves cell spreading, dissociation of cell-cell adhesions and radial cell migration (Figure 1A and 2A) [27, 28]. Most HCA7 cells did not lose their cell-to-cell contacts in response to HGF but cell colonies spread enormously in a radial fashion and eventually lost their organized structure (Figure 1B and 2B). Nevertheless, in both cell lines cloudberry extract clearly inhibited the HGF-induced morphological response (Figure 1 and 2, Supplementary Movies S3 and S4). In cloudberry-treated HCA7 cells, the increase in scatter area after 15 hours of HGF treatment was 53% lower than in control cells without cloudberry (Figure 1C). There was a significant difference between the control and cloudberry treatments in scatter area (HGF vs. HGF+Cloudberry *P*<0.001; *ANCOVA*) and scattering in time (*P*<0.001; *ANCOVA*).

**Figure 3:**
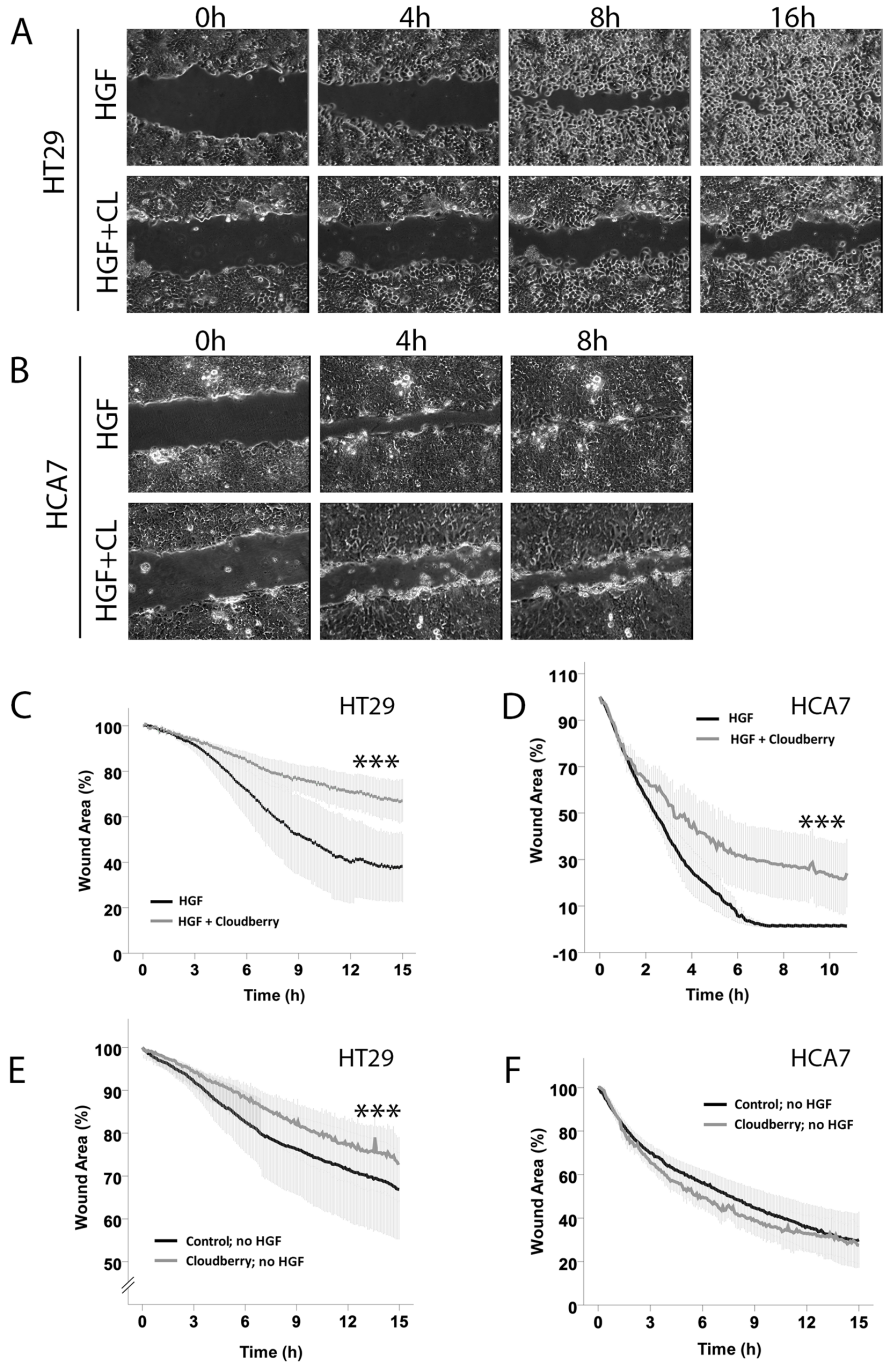
Cloudberry extract inhibits HGF-induced scratch wound closure both in HT29 and HCA7 colon adenocarcinoma cell lines, but without HGF stimulation in HT29 cells only Monolayers of **A.** HT29 and **B.** HCA7 cells were scratch wounded and the HGF-stimulated closure of the wounds was monitored every 5 min by time-lapse microscopy (Movies S5-S8 in Supplementary Materials). **C-F.** Wound areas were analyzed quantitatively every 5 min by determining the percentage of remaining wound area as compared to the area of the wound at time 0. Results (mean +/− SEM) from 3 independent experiments with HGF stimulation are shown for HT29 and HCA7 cells (**C** and **D.** *P*<0.001 (***) HGF vs. HGF+Cloudberry). Without HGF stimulation, cloudberry extract (CL) inhibited scratch wound closure in HT29 (E) *P*<0.001 (***) control vs. cloudberry) but not in **F.** HCA7 cells (mean +/− SEM from 3 independent experiments).

We used scratch wound healing assays as an alternative approach to study the effects of cloudberry extract on HGF-induced and intrinsic cell migration. HT29 cells required over 16 h for scratch wound closure (Figure 3A) which was twice the time HCA7 cells needed (Figure 3B). Evaluation of the kinetics of wound closure using time-lapse images at various time points to determine changes in wound area showed that cloudberry extract significantly inhibited HGF-induced wound healing in HT29 (Figure 3C; HGF vs. HGF+Cloudberry *P*<0.001) and HCA7 cells (Figure 3D; *P*<0.001). In both cell lines, the differences between these treatments were also significant when observed in time (Figure 3C and 3D; *P*<0.001, *ANCOVA*), demonstrating that cloudberry extract consistently slows down cancer cell migration.

Without HGF stimulation, scratch wound closure of HT29 and HCA7 cells took over 24 h. Cloudberry extract inhibited intrinsic cancer cell migration measured by scratch wound healing without HGF treatment in the *APC*-mutated HT29 cells (Figure 3E; *P*<0.001 both on average and in time) but not in HCA7 cells (Figure 3F). As APC has a well-known role in regulating directional cell migration in the gut [29] and *APC* mutations are found in the majority of sporadic colorectal cancers [30], further studies will be needed to establish whether the difference observed in intrinsic cell migration by cloudberry was indeed due to APC status or due to differences in other signaling pathways between the cell lines. Furthermore, this finding demonstrates that the effect of cloudberry in HCA7 cells was specific to HGF-induced migration. In each cell line, HGF stimulation accelerated scratch wound healing with and without cloudberry treatment (in HT29 cells, HGF vs. no HGF without cloudberry *P*<0.001 and HGF+Cloudberry vs. no HGF+Cloudberry *P*=0.016; in HCA7 cells, HGF vs. no HGF both with and without cloudberry *P*<0.001; all tested by *ANCOVA* in time). Based on these findings, we conclude that scratch wound healing in HGF-stimulated HT29 cells with cloudberry treatment resembles wound healing in these cells without HGF stimulation. Overall, since cell migration is a prerequisite for cancer progression and metastasis, our results suggest that cloudberry could slow down cancer progression by inhibiting cancer cell migration.

### Scattering and scratch wound healing in HT29 and HCA7 cells are dependent on PI3K/AKT and ERK activation

**Figure 4:**
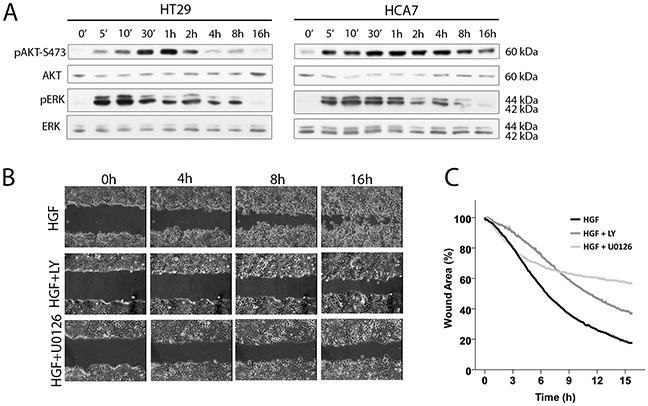
PI3K and ERK are required for HGF-induced migration **A.** HGF activates AKT and ERK in HT29 and HCA7 colon adenocarcinoma cells as shown by western blot analysis. **B.** Scratch wound healing is dependent on PI3K/AKT and ERK activation as treating the HT29 cells with the PI3K inhibitor LY294002 (LY) and the MAPKK (MEK) inhibitor U0126 prevents wound closure as determined by time-lapse microscopy. **C.** Wound areas in HGF-stimulated HT29 cells treated with LY294002 (mean from two independent experiments), U0126 (one experiment) and without inhibitors (mean from 3 independent experiments) were analyzed quantitatively every 5 min by determining the percentage of remaining wound area as compared to the area of the wound at time 0.

It is well-documented that HGF-induced cell scattering, migration, and invasion in different cell types involves downstream signaling from the Met receptor to the activation of PI3K/AKT and Ras/ERK pathways [23, 31–35]. We confirmed by western blotting for phosphorylated forms of AKT and ERK that HGF stimulation of HT29 and HCA7 cells led to sustained activation of both AKT and ERK, both of which increased by 5 min after the addition of HGF, reached a maximum level after 1 – 4 h and then gradually decreased to nearly basal levels by 16 h (Figure 4A). HT29 cells showed a biphasic activation of ERK, decreasing transiently at 30-60 min after stimulation, similar to that reported for HGF-treated mammary rat fibroblasts [33]. While there is no clear evidence for why ERK activation is biphasic, we suggest this is because of cell spreading and scattering (Figure 1), allowing integrin-induced ERK activation [36]. The PI3K inhibitor LY294002 and the MEK1 inhibitor U0126 were used to determine whether HGF-induced scratch wound closure and scattering in HT29 and HCA7 cells were, indeed, dependent on the activation of PI3K and ERK pathways. Treatment of cells together with HGF and the inhibitors resulted in partial but clear inhibition of scratch wound healing (Figure 4B and 4C for HT29 cells) and scattering in both cell lines. LY294002 inhibited HGF-induced AKT activation and U0126 inhibited ERK activation, as expected (data not shown).

### Cloudberry extract inhibits HGF-induced AKT and ERK activation in HT29 and HCA7 cells

**Figure 5:**
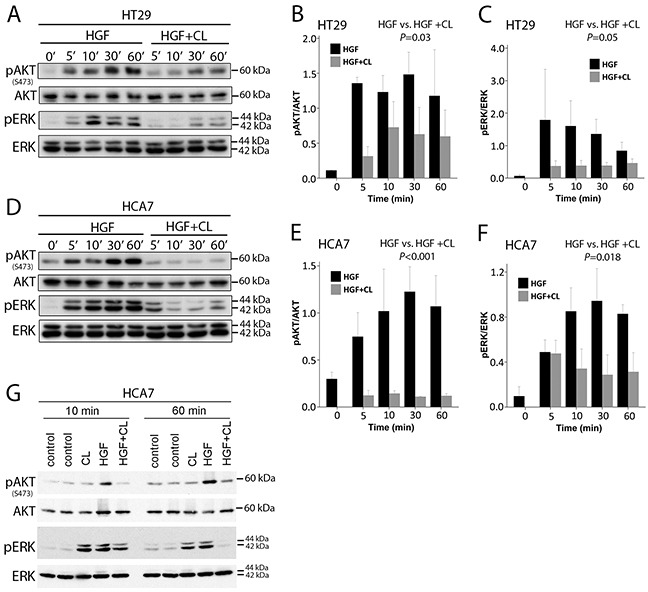
Cloudberry extract inhibits HGF-induced AKT and ERK activation **A.** HT29 and **D.** HCA7 cells were analyzed by SDS-PAGE and western blotting with antibodies against phospho-AKT(Ser473), phospho-ERK, and total AKT and ERK. Quantification of western blots of pAKT/AKT (HGF vs. HGF + CL, *P*=0.03) and pERK/ERK (*P*=0.05) levels in HT29 **B-C.** and HCA7 (**E-F.** *P*<0.001 and *P*=0.018, respectively) cells from 3 independent experiments. **G.** The effect of cloudberry extract in the absence of HGF Error bars indicate SEM.

We next studied whether the inhibitory effect of cloudberry extract on HGF-induced motility is accompanied by downregulation of PI3K and ERK pathways. Cloudberry extract inhibited HGF-induced phosphorylation of AKT and ERK in HT29 and HCA7 cells at 5 to 60 min (Figure 5A and 5D). Densitometric analyses and quantification of band intensities from three independent experiments revealed a maximum of 77% reduction in the ratio of pAKT/AKT levels (Figure 5B) and 79% reduction of pERK/ERK (Figure 5C) in HT29 cells, and a maximum of 91% reduction of pAKT/AKT (Figure 5E) and 70% reduction of pERK/ERK (Figure 5F) in HCA7 cells. Taken together, these results demonstrate that HGF-induced cell migration in HT29 and HCA7 cells is dependent on the activation of the PI3K/AKT and Ras/ERK pathways, both of which contribute to HGF/Met-induced invasive growth program in cancer cells [4], and that inhibition of the activation of these pathways by cloudberry extract explains its restricting effects on cancer cell migration. Cloudberry does not affect pAKT in the absence of HGF at either 10 min or 60 min, but as expected reduces HGF-induced AKT activation (Figure 5G). Surprisingly, cloudberry alone slightly increases pERK, but nevertheless reduces HGF-induced ERK activation. These results show that cloudberry does not reduce pERK or pAKT in unstimulated cells.

### Cloudberry extract inhibits HGF-induced Met receptor activation

**Figure 6:**
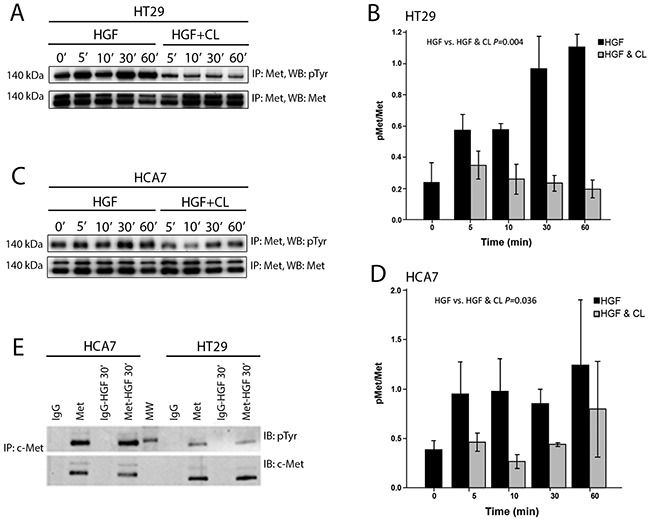
Cloudberry extract inhibits tyrosine phosphorylation of the Met receptor in HGF-treated colon adenocarcinoma cells HGF or HGF and cloudberry extract (CL) treated **A.** HT29 and **C.** HCA7 cells were lysed, immunoprecipitated with antibody against the Met receptor, and immunoblotted with anti-phosphotyrosine antibody (pTyr), followed by immunoblotting with anti-Met antibody. Quantification of western blots of pMet/Met levels in HT29 (**B.** *P*=0.004 HGF vs. HGF+Cloudberry) and HCA7 (**D.** *P*=0.036) cells from 3-4 independent experiments. Error bars indicate SEM. **E.** HT29 or HCA7 cells treated with or without HGF for 30 min were lysed, immunoprecipitated with control IgG or anti-Met antibody, and immunoblotted for anti-phosphotyrosine antibody and anti-Met antibody. Blots are representative of 3 independent experiments. MW, molecular weight markers.

We then studied whether cloudberry extract inhibits HGF-induced motility of HT29 and HCA7 at the level of activation of its receptor Met. Cells were treated with HGF and HGF together with cloudberry extract for the indicated times, followed by immunoprecipitation of Met and western blotting for tyrosine phosphorylation (Figure 6A and 6C). HGF treatment induced tyrosine phosphorylation of Met, which was inhibited by cloudberry extract as assessed by the reduction in pMet/Met levels in HT29 (Figure 6B; *P*=0.004, HGF vs. HGF+Cloudberry) and HCA7 cells (Figure 6D; *P*=0.036, HGF vs. HGF+Cloudberry). This indicates that cloudberry extract reduces HGF-induced ERK and AKT activation and cell migration by directly inhibiting activation of the Met receptor and therefore preventing receptor signaling. It is noteworthy that migratory signaling responses detected by global cell analysis (western blotting) are transient, because following the initial stimulus signals become localized only to the cells at the edge of the wound edge or cell colonies, rather than in all cells. Indeed, the wound/colony edge cells show impaired extension of lamellipodia throughout our 16-hour movies (Supplementary Movies S1-S8), indicating that the effect of cloudberry extract is not transient on migration.

Given that the effect of cloudberry extract in inhibiting HGF signaling is very rapid, acting within minutes of addition, it is possible that it acts outside the cell to inhibit HGF binding to Met or specifically inhibits Met tyrosine kinase activity. The cloudberry extract was particularly rich in ellagitannins, which are complex derivatives of ellagic acid, but it also contained moderate amounts of flavanols and phenolic acids (Table 1). Based on previous findings of the inhibitory effect of ellagic acid on cell migration [37] and the sheer abundance of ellagitannins in the cloudberry extract, we suggest that they are likely to cause Met inhibition. Ellagitannins are large molecules and therefore not capable of entering the cell, which implies that cloudberry extract interferes with the binding of HGF to the Met receptor outside the cell. However, cloudberry also contains low levels of free ellagic acid which can enter and effectively accumulate in Caco-2 intestinal epithelial cells [38], and therefore we cannot entirely exclude the possibility that cloudberry compounds act inside the cell.

**Table 1:** Concentrations of phenolic compounds in the cloudberry extract (μg/mL) and in the cell culture medium (μg/mL)

	μg/mL extract	μg/mL in cell culture medium
Ellagic acid	1067	5.3
Ellagitannins	7251	36.3
Hydroxycinnamic acids	1757	8.8
Hydroxybenzoic acids	1177	5.9
Flavanols	3822	19.1
Anthocyanins	3	0.02
Flavonols	238	1.2

Here we used an unfractionated cloudberry extract containing all compounds of the berry. The concentration of cloudberry extract in the cell culture medium was moderate, and the concentrations of ellagitannins (36 μg/mL) and total polyphenols (77 μg/mL) were in the same range as used in some previous studies [39]. However, in the absence of clinical pharmacokinetic data on the metabolism of cloudberry ellagitannins in the gut, it is difficult to estimate the accurate concentrations likely to be reached in the colon lumen *in vivo*.

### Cloudberry inhibits intestinal tumorigenesis and AKT activation in tumors of cloudberry-fed Min mice

**Figure 7:**
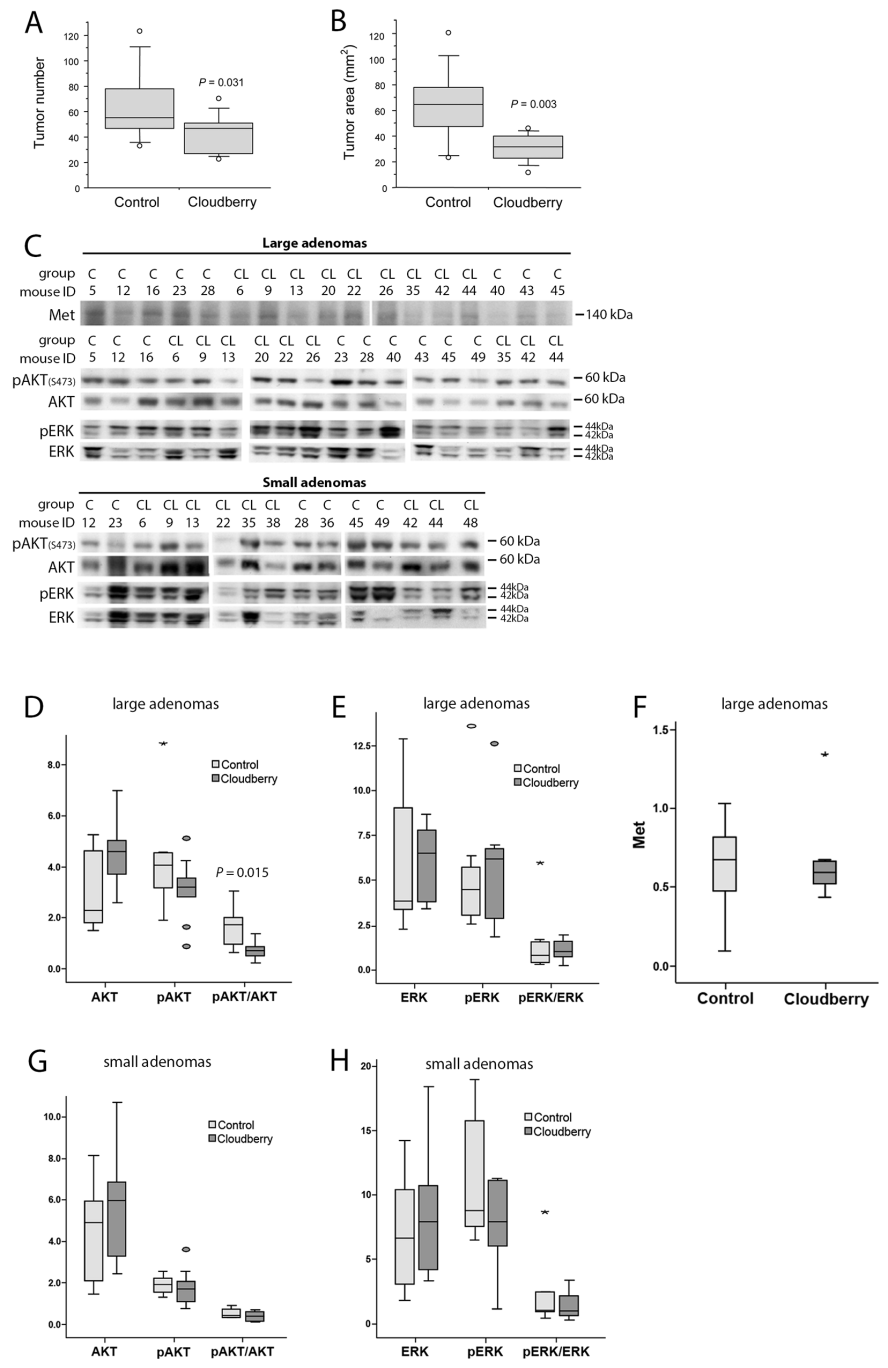
Cloudberry feeding inhibited intestinal tumorigenesis in the Min mouse **A.** The number of tumors and **B.** the tumor area (burden) in cloudberry-fed Min mice (n = 10-12 / group) were decreased in comparison to control-fed mice as reported previously [22]. **C.** Levels of Met, pAKT, AKT, pERK, and ERK in large adenomas (diameter > 1.5 mm), and levels of pAKT, AKT, pERK, and ERK in small adenomas (diameter < 1.0 mm) from each mouse in the control (C) and cloudberry-fed (CL) groups were analyzed by western blotting. **D.** The large adenomas from cloudberry-fed mice showed a significant reduction in the relation of pAKT/AKT levels. **G.** A similar but non-significant trend was seen in small adenomas of the cloudberry-fed mice. No significant differences were seen between the control and cloudberry groups either in **E, H.** ERK or in **F.** Met levels. In box and whiskers plots, a circle indicates an outlier and an asterisk (*) a far outlier.

To evaluate anti-carcinogenic effects of cloudberry *in vivo*, Min mice were fed a high-fat non-fiber diet as a control or the same diet supplemented with 10% (w/w) of freeze-dried cloudberry for ten weeks. The high fat content of the diets was chosen to better mimic current human diets in Western societies. The experiments have been described in detail elsewhere [22]. The mice in the cloudberry group produced significantly fewer intestinal tumors than the control mice (Figure 7A, *P* = 0.031; Mann-Whitney U-test). In addition, cloudberry feeding resulted in over 60% reduction in the tumor area (*P* = 0.003; Figure 7B), indicating a strong inhibitory effect on tumor growth.

**Figure 8:**
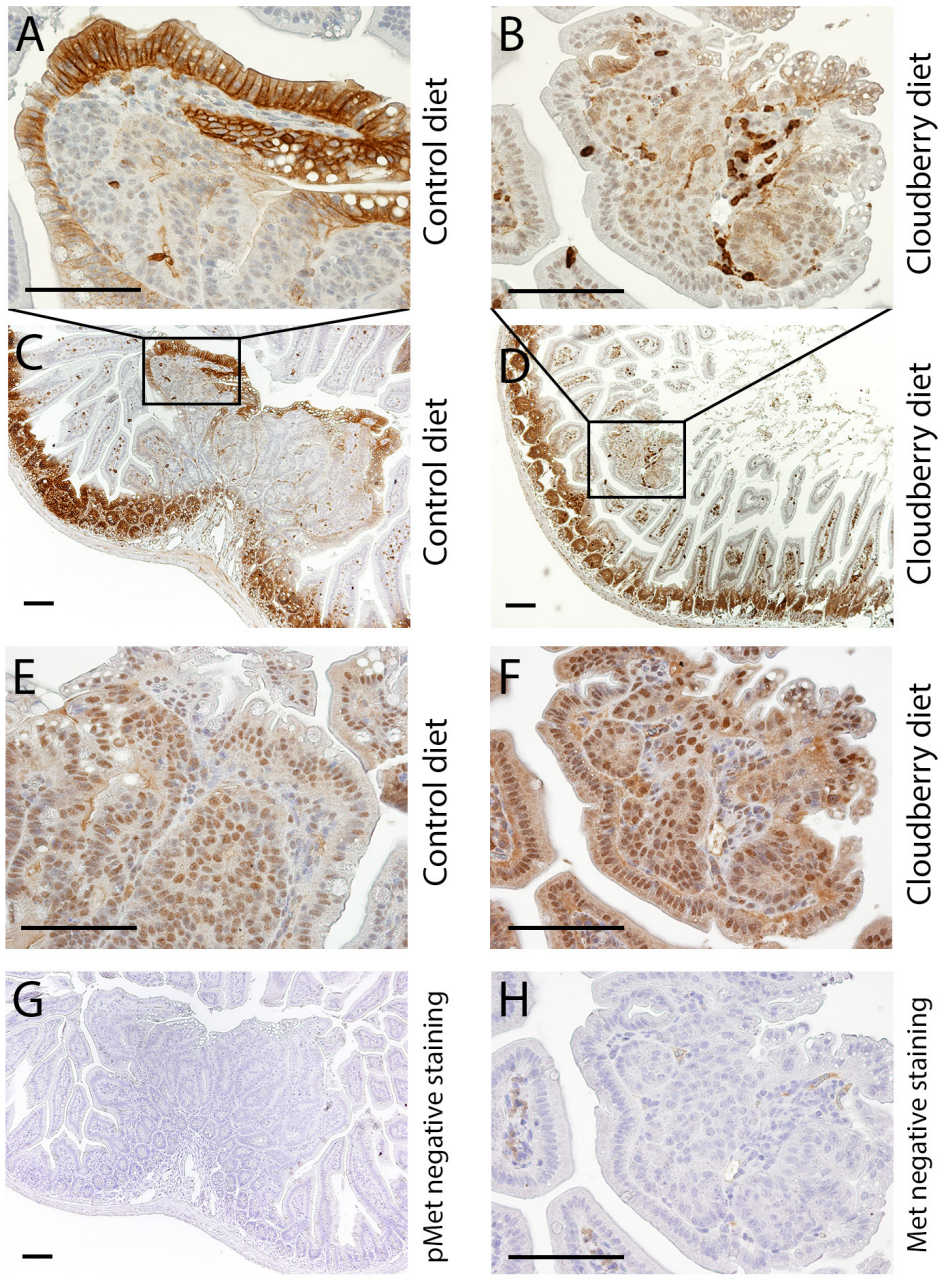
pMet is expressed and localized differently in tumors of control-fed and cloudberry-fed mice **A, C.** pMet is expressed in the edges of tumors of control-fed mice while **B, D.** only a few cells, probably lymphocytes, express pMet in the tumors of cloudberry-fed mice. The expression of total Met is shown for the tumors of **E.** control-fed and **F.** cloudberry-fed mice. **G, H.** Negative controls showed no unspecific pMet staining and its amount in the negative control of total Met was very small Bars, 100 μm.

In this study we analyzed the signaling pathways involved in Met activation in adenoma samples from the control and cloudberry-fed mice. We analyzed pAKT, AKT, pERK and ERK levels by western blotting separately in small (<1 mm) and large (>1.5 mm) diameter adenomas using a representative tumor sample from each mouse in the control (n=9) and cloudberry groups (n=9; Figure 7C). The sample per mouse was obtained by excising all tumors from the intestine and pooling them according to the size category. The resulting bands were quantified by densitometric analyses and the ratio between the phosphorylated form and total AKT and ERK levels was used as a measure of their activation status. Large adenomas of cloudberry-fed mice showed a significant reduction in the ratio of pAKT/AKT in comparison to adenomas from the control mice (Figure 7C and 7D; *P*=0.015, Mann-Whitney U test), and a similar but non-significant trend was seen in small adenomas (Figure 7C and 7G). No differences in ERK activation was found either in small (Figure 7H) or large diameter adenomas (Figure 7E). We also analyzed total Met levels in large adenomas but found no differences between the dietary groups (Figure 7C and 7F) even though the variation in Met levels was notably smaller in the cloudberry-fed group (Figure 7F). As we were not able to reliably determine pMet levels in the adenoma by western blotting, we utilized paraffin-embedded intestinal tissue samples collected from four control and seven cloudberry-fed mice to stain pMet and Met by immunohistochemistry (Figure 8). Adenomas from control mice had a strong positive staining of pMet at the edge of tumors (Figure 8A and 8C) whereas in cloudberry-fed mice pMet predominantly localized in the crypt area of the mucosa (Figure 8D; as also observed in control mice), not in the adenoma (Figure 8B). Unlike in human colorectal cancer, in which Met is clearly expressed [8], one earlier study [40] found no Met overexpression in tumors of Min mice and Met levels in general were near the detection limit in adult Min mouse gut. pMet was not measured, and thus they did not analyze Met activity [40].

In line with the effects of cloudberry extract on colon adenocarcinoma cells *in vitro*, cloudberry is likely to have a local effect on cellular responses relevant to cell invasion at the edge of tumors as demonstrated by our pMet results. This would be more relevant for larger tumors that are beginning to invade the surrounding tissues even though there was a trend for reduced AKT activation in small adenomas, indicating that changes start occurring during early stages of tumorigenesis. Our results suggest that inhibition of the Met and PI3K/AKT pathways are involved in mediating the anti-carcinogenic effects of cloudberry *in vivo*. Using Affymetrix microarray, we have observed a six-fold increase in HGF expression in the normal appearing mucosa of the Min mouse in comparison to the mucosa of wild-type C57BL/6J mouse (unpublished observation), indicating a role of HGF/Met signaling in intestinal tumor development in the Min mouse, similar to human studies. Importantly, a recent study demonstrated that stromal-derived HGF and consequent Met activation is needed to maintain stem cell-like properties of colon cancer cells through activation of Wnt-β-catenin-dependent transcription [41], suggesting an active role for tumor microenvironment and HGF/Met signaling possibly early on in the development of colorectal cancer. As dietary factors have been shown to alter the activity of both Wnt and Met pathways [22, 42], regulating the tumor microenvironment by dietary means provides an intriguing target for cancer prevention.

## CONCLUSIONS

Our results with the Min mouse demonstrate that cloudberry is able to inhibit the early stages of colon carcinogenesis [22]. This mouse strain is the most widely used animal model for colon cancer studies, but it does not measure metastasis, since metastases do not form. Our results with colon carcinoma cells may better predict the capacity of cloudberry to inhibit later stages of colon carcinogenesis, when cancer cells become more invasive. Indeed, we show here using cell scattering and scratch wound healing assays that cloudberry extract strongly inhibits HGF-induced and Met receptor-mediated cancer cell migration, a prerequisite for cancer cells to metastasize. Significantly, we also demonstrate that this is mediated by cloudberry's ability to inhibit the phosphorylation and thus activation of the Met receptor and its downstream activation of both PI3K/AKT and ERK pathways. Since these pathways are known to drive the invasive growth program in cancer cells [4], cloudberry extract could act to slow cancer progression and spreading. As the Met receptor is recognized to be one of the major therapeutic targets of the hallmarks of cancer [43], dietary phytochemicals acting as natural HGF/Met inhibitors are likely to have therapeutic value in preventing cancer invasion and metastasis.

We suggest that the chemopreventive effects of cloudberry are mainly due to ellagitannins, the main class of its polyphenols, even though it also contains flavanols, organic acids and some free ellagic acid. It is, however, possible that synergistic effects of the phenolic compounds occur and they together may exert a stronger chemopreventive effect than any of the compounds alone [39]. Major ellagitannins in cloudberry are sanguiin H6, sanguiin H10 and lambertinian C [44]. A similar profile of ellagitannins is found in raspberries, while other good sources of ellagitannins include blackberry, arctic bramble, strawberry, pomegranate, certain nuts, and oak-aged wines [44, 45]. Of these, pomegranate is the most studied source of ellagitannins, which together with ellagic acid and its metabolites, have previously been shown to have anti-carcinogenic activity in cell cultures and animal models [46]. Pomegranate ellagitannins have recently been studied in clinical trials for treatment of prostate cancer [47]. So far, an effect of berries in cancer treatment has been tested for black raspberry in two phase 1 studies in FAP and colorectal cancer patients with promising results [19, 20], and our results suggest that cloudberry should also be tested in the future.

In summary, we show here that cloudberry extract inhibits HGF-induced scattering and migration into a scratch wound in HT29 and HCA7 colon adenocarcinoma cells, which is accompanied by inhibition of Met receptor activation and downstream signaling to the PI3K/AKT and ERK pathways. Furthermore, we found that AKT activation and localization of pMet on tumor edges was reduced in large adenomas from cloudberry-fed Min mice compared to control mice. We have previously reported that cloudberry feeding results in inhibition of nuclear accumulation of β-catenin and cyclin D1 in the Min mouse tumors as well as reduction in expression of *e.g.* prostaglandin E receptor subtype EP4 in the normal appearing mucosa [22], so presumably there are several mechanisms contributing to the inhibitory effect of cloudberry on tumorigenesis. Taken together, these results suggest that cloudberry and its compounds possess strong chemopreventive activity against colon cancer which is at least partly mediated through the inhibition of the Met receptor and downstream signaling to the PI3K pathway.

## MATERIALS AND METHODS

### Reagents and antibodies

Human fibronectin, U0126 and fluorescein isothiocyanate(FITC)-conjugated phalloidin were purchased from Sigma-Aldrich (Gillingham, UK), LY294002 from Merck, recombinant human HGF from R&D Systems (Abingdon, UK), antibodies against pAKT [Ser473], AKT and pERK from Cell Signaling Technology, ERK1 (K-23), Met (C-12, SP260), protein A/G PLUS-agarose were from Santa Cruz Biotechnology (Santa Cruz, CA), and Immobilon P membrane from Millipore.

### Preparation of cloudberry extract for cell culture studies

Six grams of freeze-dried cloudberries were crushed (including the seeds and peels) and extracted twice with 25 mL of methanol by vortexing for 1 min followed by centrifugation for 10 min at 3500*g*. The supernatants were combined and methanol was removed by rotary evaporation at 30°C under vacuum. The semidried sample was reconstituted in 4.5 mL of Milli-Q water and frozen. Two independent preparations of cloudberry extract were used in the study. The phenolic profile of cloudberry extract was analyzed as described below and the concentrations of phenolic compounds in the cloudberry extract and cell culture medium are shown in Table 1.

### UPLC analysis of phenolic profile of cloudberry extract

Preparation of extract from freeze-dried cloudberries followed the same procedure as for the cell culture studies. The final cloudberry extract was dissolved in 20% methanol, and filtered with 0.2 μm syringe filters prior to analysis. The phenolic profile of berry extract was determined according to the method described earlier [48] using Waters ACQUITY ultra performance liquid chromatography (UPLC) coupled with eλ photodiode array (PDA) and fluorescence (FLR) detectors. The column used was a Waters HSS T3 C18, 1.7 μm, 2.1 × 150 mm, with 5 μL injection volume, and separation was achieved using a gradient program with water/0.5% formic acid and acetonitrile/0.5% formic acid with a constant flow rate of 0.5 mL/min. Samples were analysed in triplicate with double injections. Identification of phenolic compounds was based on their UV spectra, and phenolics were clustered into seven subclasses: ellagic acids, 365 nm and ellagitannins as ellagic acids, 280 nm; anthocyanins as cyanidin-3-glucosides, 520 nm; flavonols as rutin, 365 nm; hydroxycinnamates as chlorogenic acid, 320 nm; hydroxybenzoates as gallic acid, 280 nm; catechins and flavan-3-ols as procyanidin B2, FLR.

### Cell culture

The HT29 (purchased from American Type Culture Collection for this study) and HCA7 human colon adenocarcinoma cells (kindly provided and authenticated by Walter Bodmer, University of Oxford; [49]) were cultured at 37°C in a 5% CO_2_ atmosphere in DMEM (Gibco-Invitrogen) supplemented with 10% FCS, penicillin and streptomycin. For all experiments, cells were seeded on fibronectin-coated (10 μg/mL) tissue culture dishes. The concentration of cloudberry extract applied to cells was chosen based on preliminary experiments where the effects of concentrations ranging between 0 - 20 μL/mL were tested on cells for 24 - 48 h. The chosen concentration of 5 μL/mL did not result in reduction in cell numbers nor induce cell necrosis as assessed using a hemocytometer and trypan blue staining (data not shown).

### Analysis of cell migration using time-lapse microscopy

For cell scattering experiments, HT29 (3×10^4^/mL) and HCA7 (6×10^4^/mL) cells were seeded on fibronectin-coated dishes containing a 13-mm glass coverslip in the center and incubated for 48 - 72 h before time-lapse microscopy. Cells were treated either with 40 ng/mL HGF or HGF together with 5 μL/mL of cloudberry extract in growth medium. Cloudberry extract was applied to cells 30 min before HGF. During the time-lapse experiments cells were incubated at 37°C in a humidified chamber at 5% CO_2_. Phase-contrast images of live cells were collected every 5 min for 16 hours with Tempus software (Andor Technology, Belfast, UK), using a KPM1E/K-S10 CCD camera (Hitachi, Denshi, Japan) mounted on an Axiovert microscope using a 20X Plan/Neofluar objective (Zeiss). For wound healing experiments, cells were seeded at high density on fibronectin-coated 35-mm-diameter tissue culture plastic dishes and allowed to grow to 100% confluence. Cells were washed twice with PBS (phosphate buffered saline) without calcium and magnesium (Gibco-Invitrogen), scratch wounded with a 10 μL pipette tip, rinsed twice with medium and treated either with 40 ng/mL HGF or HGF together with 5 μL/mL of cloudberry extract in growth medium. Wound closure was monitored by time-lapse microscopy as described for cell scattering experiments.

### Quantification of wound and scatter areas in time-lapse images

Wound healing images were analyzed quantitatively at various time points by determining the percentage of remaining wound area as compared to the area of the wound at the first time point. The wound area at each time point was measured with the geodesic active contours [50] by setting a few initial circular contours in the middle of the wound, and making the contours iteratively propagate to fill the whole wound area. As the active contours can merge and split during the iterative process, final area was defined by the outermost contour to exclude effect of all inner contours formed especially in cloudberry images. All processing was carried out with BioImageXD software, an open access, high-throughput image-processing computer platform [51]. The scatter area evolution was measured as wound area except only the single initial contour, which included whole scatter area, was used. The contour was made to contract during iterations after which the area was defined as a combination of all isolated contours generated during the contraction of the initial contour. All contours were included in area analyses at all the time points as these merged to one larger area during the experiment.

### Whole cell extracts and western blot analyses

HT29 (3×10^4^/mL) and HCA7 (6×10^4^/mL) cells were seeded on fibronectin-coated tissue culture plastic and incubated for 48-72 h. Cells were treated either with 40 ng/mL HGF or HGF and 5 μg/mL of cloudberry extract in serum-free (HT29) or low-serum (HCA7) DMEM for the indicated times. Cells were washed twice with ice-cold PBS and lysed for 10 minutes on ice in lysis buffer (50 mmol/L Tris-HCl (pH 7.4), 150 mmol/L NaCl, 1 mmol/L EDTA, 1% Triton X-100, 1 mmol/L PMSF, 10 μg/mL leupeptin, 2 μg/mL aprotinin, 1 mmol/L dithiothreitol, 1 mmol/L NaF, and 0.2 mmol/L Na_3_VO_4_). Lysates were clarified by centrifugation at 14 000 × *g* for 10 min at 4°C. Protein concentrations were determined using BioRad Bradford protein assay reagent. Lysates were denatured in Laemmli sample buffer and heated at 98°C for 5 min. Twenty μg of protein from the extracts was resolved on 10% SDS-PAGE and transferred to PVDF membrane (Immobilon P, Millipore). Membranes were blocked in 5% bovine serum albumin (pAKT, pERK) or 5% non-fat milk (AKT, ERK) in Tris-buffered saline and 0.1% Tween-20 for 1 h and probed with primary antibodies overnight at 4°C (pAKT and pERK) or for 2 h at room temperature (AKT, ERK). After extensive washing, membranes were incubated with horseradish-peroxidase-conjugated secondary antibody for 1 h at room temperature and developed using enhanced chemiluminescence kit (Amersham/GE Healthcare, UK). Band intensities were determined and quantified using a GS-800 densitometer and Quantity One software (BioRad, Hercules, CA). Blots were stripped between determination of phosphorylated and total protein levels for AKT and ERK. Small diameter (<1 mm) and large diameter (>1.5 mm) adenomas of Min mice were collected separately and analyzed for Met, pAKT, AKT, pERK, and ERK as described above.

### Immunoprecipitation

Cells were seeded on 100-mm-diameter fibronectin-coated tissue culture dishes, grown to semi-confluency, and treated either with HGF or HGF and cloudberry extract. Cell lysates were prepared as described above except that cells were lysed with 1000 μL of lysis buffer per sample, containing 10% of glycerol. 250 μg of protein from the lysates were incubated with 25 μL of anti-Met antibody conjugated to protein A/G PLUS-agarose (Santa Cruz) for 4 h at 4°C. The pellets were collected by centrifugation at 1000 × *g* and washed four times for 5 min each with lysis buffer at 4°C. The pellets were resuspended in 30 μL of Laemmli sample buffer, heated at 98°C for 5 min, resolved on 7.5% SDS-PAGE and transferred to PVDF membrane. The blots were probed with anti-phosphotyrosine antibody (Clone 4G10, Upstate), followed by stripping and probing for total Met levels with anti-Met antibody (Santa Cruz). The blots were developed and bands quantified as described above.

### Actin staining for confocal microscopy

HT29 and HCA7 cells were seeded on fibronectin-coated glass coverslips, incubated and treated with HGF and cloudberry extract as described above. Cells were fixed in 4% paraformaldehyde in PBS for 20 min, permeabilized in 0.2% Triton-X-100 for 5 min, and stained with FITC-conjugated phalloidin to visualize actin filaments. Cells were mounted in Dako fluorescent mounting medium (DAKO, Ely, UK). Images were collected with LSM510 software, using a confocal laser scanning microscopy (LSM510, Carl Zeiss, Welwyn Garden City, UK) and a 40X/1.3 NA Plan Neofluar objective.

### Animals and diets

The Laboratory Animal Ethics Committee of the University of Helsinki, Finland, approved the study protocol. Research was done complying with good laboratory practice, including national and institutional guidelines for the care and use of animals. Male and female C57BL/6J-*Apc^Min^*/J (Min) mice (n=10-12/group) were fed AIN93-based high-fat control diet or the same diet supplemented with 10% (w/w) freeze-dried cloudberry for ten weeks as described previously [22]. The high fat content (20% w/w corresponding 40% of energy intake) of the diets was chosen to better mimic current human diets in Western societies. The number and size of intestinal adenomas were determined blind by two observers, using a dissecting microscope connected to a screen with magnification of 67 by two observers blind to treatment. Tumors were excised, snap frozen in liquid nitrogen and stored at −70°C until further analyses.

### Immunohistochemical staining

Paraffin tissue sections were deparaffinized and subjected to heat-induced antigen retrieval in microwave oven for 15 min at 800W power in 250 ml of citrate buffer (pH 6.0), followed by a 20 min cooling period. Immunostaining with anti-Met (c-Met, ab59884; 1:400), anti-phospho-Met (ab5662, c-Met, phospho-Y1230 + Y1234 + Y1235; 1:1500) (both antibodies from Abcam, Cambridge, UK), and counterstaining with Mayer's hemalaum (Merck) were performed using UltraVision Quanto Mouse on Mouse HRP detection kit (LabVision/Thermo Fisher Scientific, Cheshire, UK) for Met, and BrightVision Poly-HRP-anti rabbit detection kit (Immunologic, Duiven, Netherlands) for phospho-Met. Negative control tissues were prepared similarly, except the primary antibody was replaced with normal antibody diluent (Immunologic, Duiven, Netherlands) for anti-Met and diluent containing 10% goat serum for anti-phospho-Met. The immunostained sections were imaged using a Leica DM4000 microscope (Wetzlar, Germany) and an Olympus DP70 camera (Tokyo, Japan).

### Statistical analyses

Statistical analyses were carried out using StatView 5.0.1 (SAS Institute Inc., Cary, NC) and PASW 18.0.1 (SPSS Inc., 2009, Chicago, IL) softwares. Differences of standardized wound and scatter-data behavior in treatment groups (HGF vs. HGF + cloudberry) in time were compared with parametric ANCOVA (group*time) interaction terms. The binary presence of HGF and type of cell (HCA7 and HT29) was also used as a stratifying variable, or as a group variable instead of cloudberry treatment in the analyses. Linear fit was utilized; it suited the data set best both statistically and contextually. The western blot results were analyzed by Mann-Whitney U test and differences were considered significant at *P* < 0.05.

## SUPPLEMENTARY MOVIES
















