# Supplementary material for: Ellagitannin-rich cloudberry inhibits hepatocyte growth factor induced cell migration and phosphatidylinositol 3-kinase/AKT activation in colon carcinoma cells and tumors in Min mice
Source: Oncotarget. 2016 May 30;7(28):43907–23. doi: 10.18632/oncotarget.9724 (PMC5190067; doi:10.18632/oncotarget.9724)
Supplement: Supplementary file 1 [file oncotarget-07-43907-s001.pdf]

SUPPLEMENTARY MOVIES

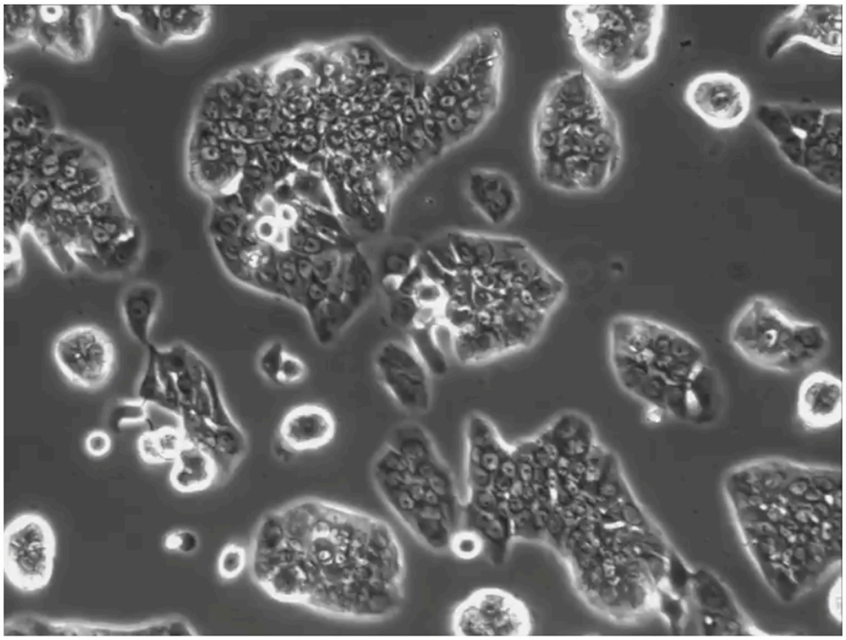

Supplementary Movie S1: HGF-induced scattering in HT29 cells.

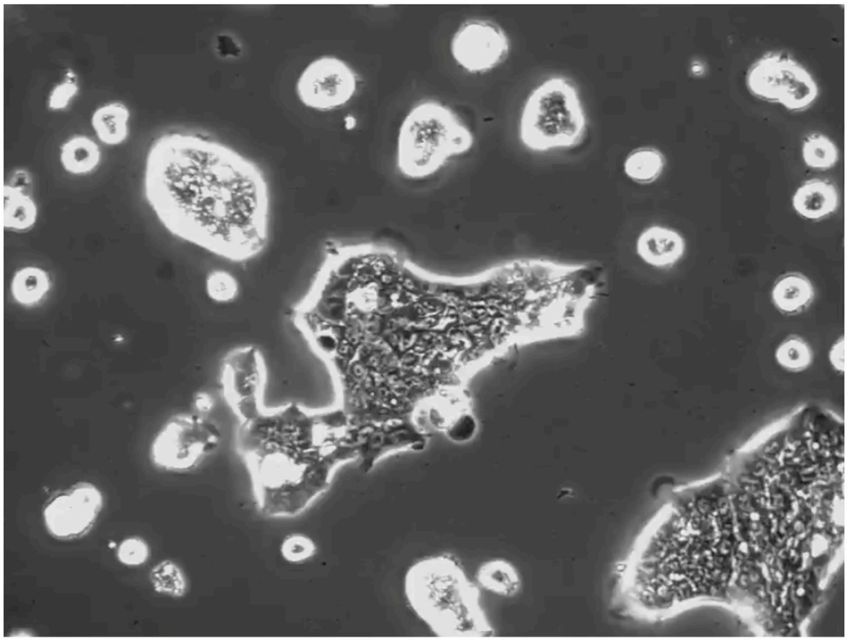

Supplementary Movie S2: HGF-induced scattering in HCA7 cells.

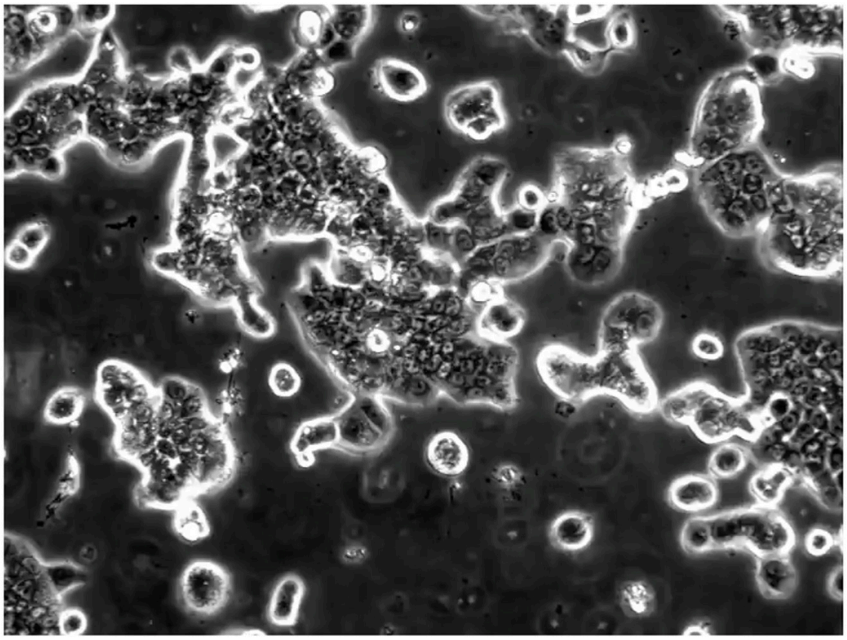

Supplementary Movie S3: HGF-induced scattering in cloudberry treated HT29 cells.

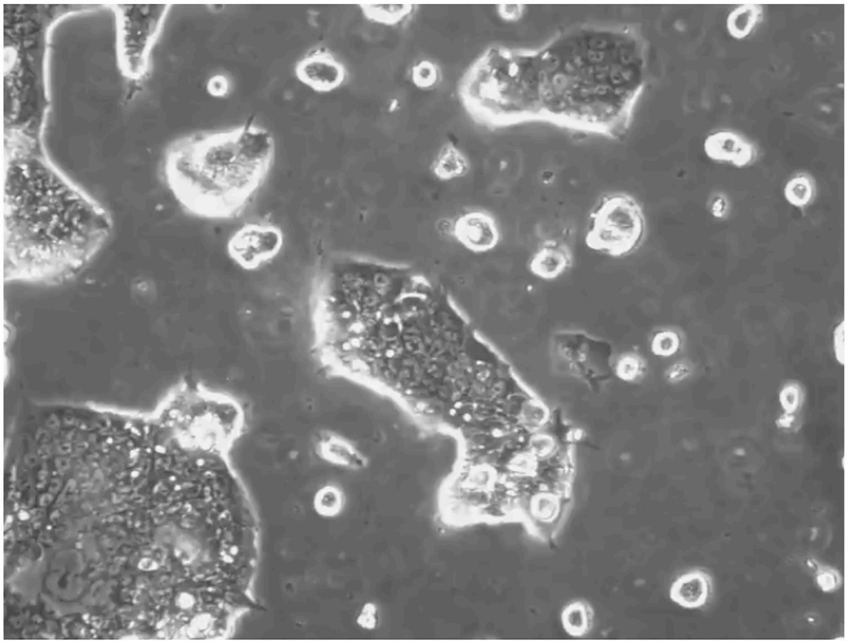

Supplementary Movie S4: HGF-induced scattering in cloudberry treated HCA7 cells.

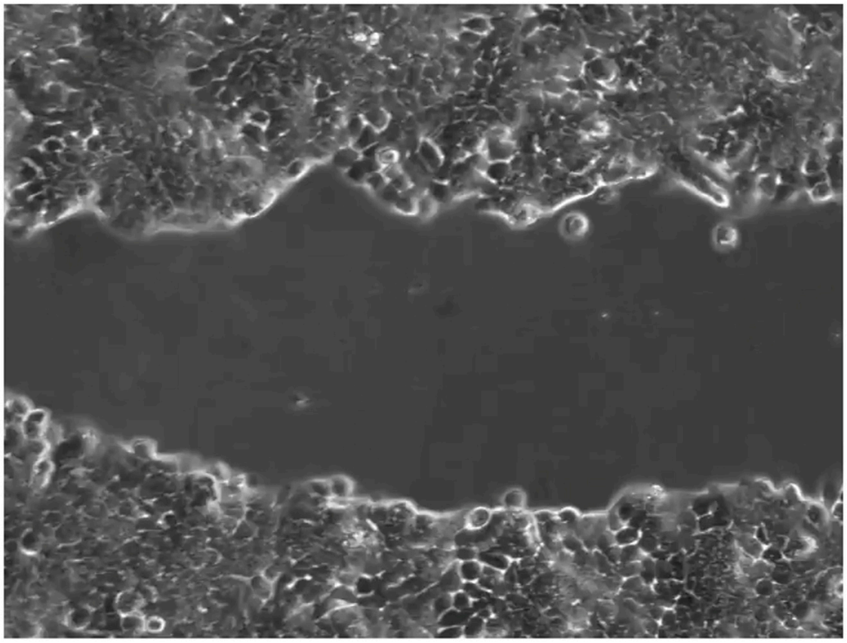

Supplementary Movie S5: HGF-induced scratch wound closure in HT29 cells.

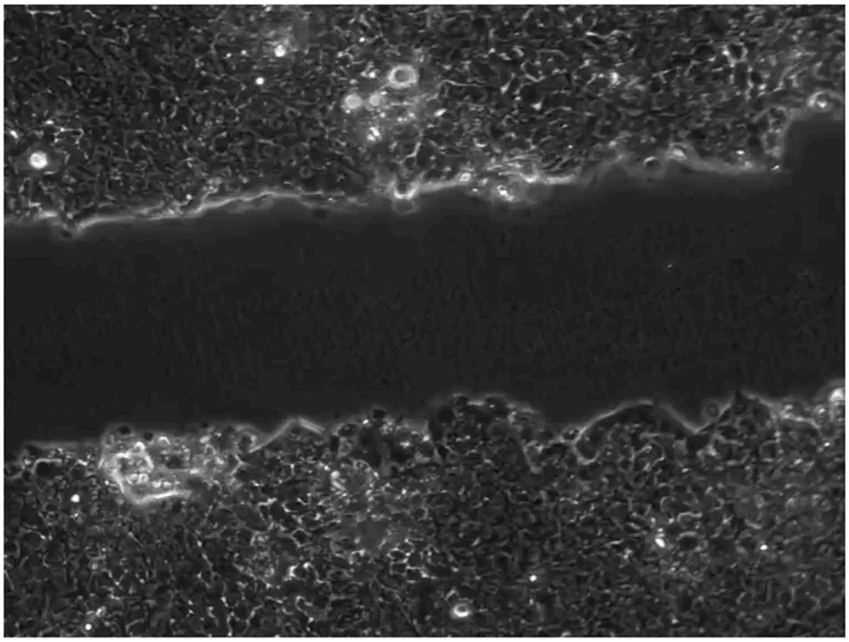

Supplementary Movie S6: HGF-induced scratch wound closure in HCA7 cells.

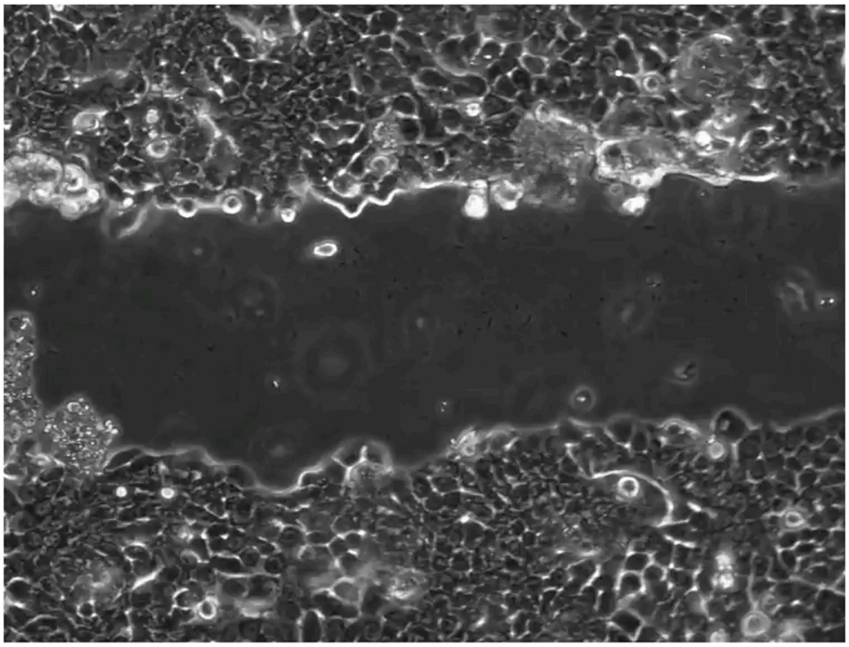

Supplementary Movie S7: HGF-induced scratch wound closure in cloudberry treated HT29 cells.

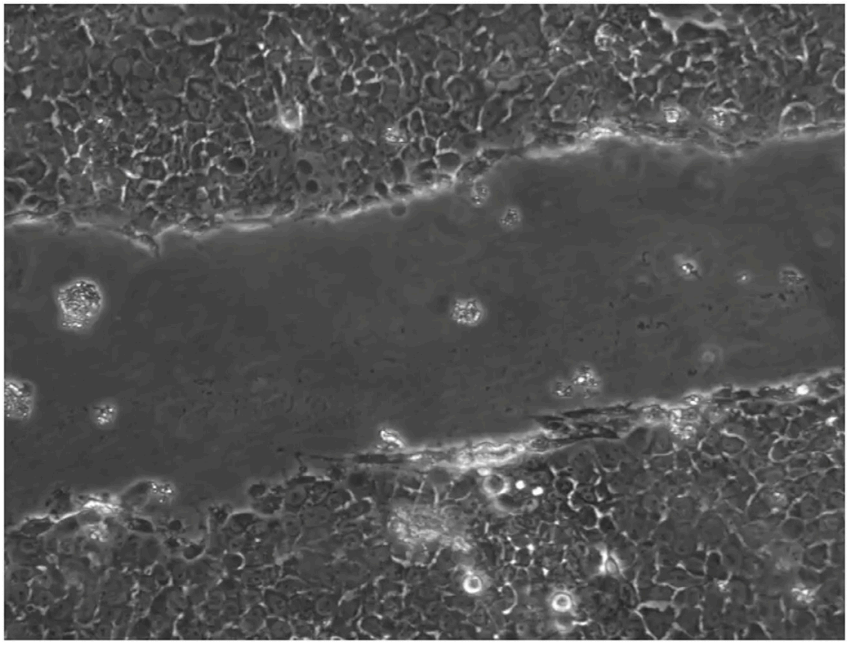

Supplementary Movie S8: HGF-induced scratch wound closure in cloudberry treated HCA7 cells.
